# BMSC-Derived Small Extracellular Vesicles Induce Cartilage Reconstruction of Temporomandibular Joint Osteoarthritis *via* Autotaxin–YAP Signaling Axis

**DOI:** 10.3389/fcell.2021.656153

**Published:** 2021-04-01

**Authors:** Yingnan Wang, Miaomiao Zhao, Wen Li, Yuzhi Yang, Zhenliang Zhang, Ruijie Ma, Mengjie Wu

**Affiliations:** The Affiliated Hospital of Stomatology, School of Stomatology, Zhejiang University School of Medicine, Key Laboratory of Oral Biomedical Research of Zhejiang Province, Hangzhou, China

**Keywords:** temporomandibular joint osteoarthritis (TMJ-OA), bone marrow mesenchymal stem cell (bMSC), autotaxin (ATX), hippo signaling pathway, cartilage reconstruction, small extracellular vesicles (sEVs), yes-associated protein (YAP)

## Abstract

**Background:** Temporomandibular joint osteoarthritis (TMJOA) seriously affects the health of patients, and the current treatments are invasive and only used for advanced cases. Bone marrow mesenchymal stem cell (BMSC)-derived small extracellular vesicles (BMSC-sEVs) may represent a safer and more effective treatment, but their role in TMJOA has not been elucidated. This study attempted to analyze the cartilage reconstruction effect of BMSC-sEVs on TMJOA and the mechanism underlying this effect.

**Methods:** BMSC-sEVs were isolated and purified by microfiltration and ultrafiltration and were subsequently characterized by nanoparticle tracking analysis, electron microscopy, and immunoblotting. TMJOA models were established *in vivo* and *in vitro*, and hematoxylin–eosin staining, immunohistochemistry, and histological scoring were performed to analyze the histological changes in TMJOA cartilage tissues treated with BMSC-sEVs. The proliferation, migratory capacity, and cell cycle distribution of TMJOA cartilage cells treated with BMSC-sEVs were detected. Furthermore, the related mechanisms were studied by bioinformatic analysis, immunoblotting, and quantitative PCR, and they were further analyzed by knockdown and inhibitor techniques.

**Results:** The acquisition and identification of BMSC-sEVs were efficient and satisfactory. Compared with the osteoarthritis (OA) group, the condylar tissue of the OA group treated with BMSC-sEV (OA^sEV^) showed an increase in cartilage lacuna and hypertrophic cartilage cells in the deep area of the bone under the cartilage. Significantly upregulated expression of proliferating cell nuclear antigen and cartilage-forming factors and downregulated expression of cartilage inflammation-related factors in OA^sEV^ were observed. In addition, we found higher rates of cell proliferation and migratory activity and alleviated G1 stagnation of the cell cycle of OA^sEV^. Autotaxin was found in the BMSC-sEVs, and key factors of the Hippo pathway, Yes-associated protein (YAP), phosphorylated Yes-associated protein (p-YAP), etc. were upregulated in the OA^sEV^ group. Treatment with BMSC-sEVs after autotaxin knockdown or inhibition no longer resulted in expression changes in cartilage-forming and inflammation-related factors and key factors of the Hippo pathway.

**Conclusions:** These results suggest that the autotaxin–YAP signaling axis plays an important role in the mechanism by which BMSC-sEVs promote cartilage reconstruction in TMJOA, which may provide guidance regarding their therapeutic applications as early and minimally invasive therapies for TMJOA, and provide insight into the internal mechanisms of TMJOA.

## Background

Temporomandibular joint osteoarthritis (TMJOA) is when the temporomandibular joint disorder (TMD) progresses to a condition marked by severe histological damage, with early signs of hard tissue changes such as cartilage absorption on the surface of the joint, osteophyte formation, shifting or perforation of the joint disc, and even facial asymmetry or mandibular retrognathism (Kim et al., 2019). The current treatment methods for TMJOA are mostly focused on treating advanced stages of the condition by applying invasive treatments, so how to achieve early and minimally invasive treatment for TMJOA needs to be addressed. In recent years, treatments such as tissue engineering therapy, immunology, and gene therapy have emerged (Abouelhuda et al., 2018; Wu et al., 2021). These biotherapies are a good attempt, but their efficacy needs to be further explored, and their mechanisms of action are not yet clear.

Currently, novel treatments for TMJOA are mostly focused on the application of cells, such as mesenchymal stem cells (MSCs), which can reduce cell apoptosis and participate in immune regulation. This approach has become a new potential treatment for joint defects and osteoarthritis (OA)-related damage. Among them, bone marrow MSCs (BMSCs) play an important role in the repair of OA and bone fractures. Compared with extraskeletal MSCs, BMSCs are superior in phenotype, morphology, function, and potential therapeutic applications (García-García et al., 2015). It has been found that BMSCs implanted in TMJOA inhibit cartilage degradation and repair diseased tissues (Chen et al., 2013; Lu et al., 2015). Thus, BMSCs have a therapeutic effect on TMJOA and can promote the reconstruction of the cartilage. However, there are some limitations to the treatment of TMJOA with MSCs, such as unevenness and a tumorous tendency, and it is reported that MSCs exert their effects mainly through secretion function (Yu et al., 2014). Given all these, more stable and reliable methods to treat TMJOA with MSCs are necessary.

Extracellular vesicle (EV) is a kind of vesicle secreted by all cell types, with diameters of approximately 50–1,000 nm that contain and transport functional contents. Among them, vesicles with diameters of roughly 50–100 nm are defined as small extracellular vesicles (sEVs), frequently classified as “exosomes,” “microvesicles,” etc. (Théry et al., 2018). They are considered to be important regulatory factors for intercellular communication and are involved in multiple pathological processes (Wang et al., 2018). Considering their small size, good targeting, satisfactory stability, and capability of crossing barriers, avoiding degradation and transporting their cargos into the cytoplasm, sEVs have become a new treatment approach to many kinds of diseases. Although it is known that MSC-derived sEVs (MSC-sEVs) exert many therapeutic applications (Witwer et al., 2019), the role of BMSC-sEVs in TMJOA has been rarely reported, which still remains unclear.

Therefore, the purpose of this study was to explore the role and molecular mechanism of BMSC-sEVs in enhancing cartilage reconstruction in TMJOA and to provide theoretical support for the clinical applications and further mechanistic interpretations of MSC-derived sEV therapy for TMJOA in the future.

## Materials and Methods

### Cell Culture

Human BMSCs were kindly provided by the Stem Cell Bank, Chinese Academy of Sciences, harvested from healthy people and cultured in Dulbecco’s minimum essential medium (DMEM; BasalMedia, China). Mandibular condylar chondrocytes (MCCs) were extracted from the condyles of the temporomandibular joints (TMJs) of New Zealand rabbits and cultured in DMEM. All media were supplemented with 10% fetal bovine serum (FBS, Yeasen, China) and 1% penicillin-streptomycin (BasalMedia, China), and all cells were cultured in a humidified incubator (Thermo Fisher Scientific, United States) with 5% CO_2_ at 37°C.

### Small Extracellular Vesicle Isolation and Purification

First, sEVs were isolated from the medium supernatant of BMSCs without FBS or penicillin-streptomycin, which was harvested after 48 h of culture. After centrifugation (4,000 rpm, 10 min), the supernatant was sequentially minifilter through polyvinylidene fluoride (PVDF) membrane filters at 450 nm (Merck Millipore, Germany) and then 200 nm (PALL, United States). Then, the flow-through was ultrafiltered by 100-kD centrifugal filter devices (Merck Millipore, Germany). A 15-ml device is usually used, and a 0.5-ml device can be used to further concentrate the sEVs, which can then be harvested by reverse centrifugation. Finally, sEVs isolated from approximately 80-ml medium supernatant were suspended in 1 × phosphate buffer saline (PBS) buffer or culture medium through the use of 0.5-ml centrifugal filter devices (Merck Millipore, Germany) for further experiments.

### Nanoparticle Tracking Analysis

For particle size and concentration determination, nanoparticle tracking analysis (NTA) was performed with a NanoSight NS300 (Malvern, United Kingdom) equipped with fast video capture and NTA analytical software. Nanoparticles were illuminated by the laser, and their movements under Brownian motion were captured for 60 s. Videos were analyzed by the software to provide the nanoparticle concentration and size distribution profiles.

### Transmission Electron Microscopy

The BMSC-sEV samples were added onto a piece of copper grid for 1 min. Then, a drop of 2% uranyl acetate was added onto the copper grid for 1 min. After drying for 10 min, the cells were examined under a transmission electron microscope (Thermo Fisher Scientific, United States).

### Immunoblotting

BMSC-sEVs and cells were lysed with radioimmunoprecipitation assay (RIPA) lysis buffer (Yeasen, China) containing protease inhibitors. The lysates were boiled with Protein SDS-PAGE Loading Buffer (GenScript, China), electrophoresed through 4–20% polyacrylamide gels (GenScript, China) and transferred onto 0.45-μm PVDF membranes (Absin, China). The membranes were blocked using 5% skim milk (Yeasen, China) in PBS. Antibodies against the following proteins were used for immunoblotting (IB) analysis: CD81 (SBI, Japan), CD63 (SBI, Japan), Ras-related protein 5 (Rab5; Biovision, United States), ALG2-interacting protein (Alix; Thermo Fisher Scientific, United States), glucose-regulated protein 94 (GRP94; Thermo Fisher Scientific, United States), aggrecan (ACAN; Bioss, China), SRY-related high-mobility group box 9 (SOX9; Bioss, China), matrix metalloproteinase 13 (MMP13; Bioss, China), RUNX family transcription factor 2 (RUNX2; Bioss, China), Collagen I (Bioss, China), proliferating cell nuclear antigen (PCNA; Proteintech, United States), cartilage-forming factors-type II collagen (Col-II; Novus, United States), Autotaxin (Abcam, China), Yes-associated protein (YAP; Absinthe, China), phosphorylated Yes-associated protein (p-YAP; LifeSpan BioSciences, United States), RhoA (Absinthe, China), large tumor suppressor kinase 1 (LATS1; Absinthe, China), large tumor suppressor kinase 2 (LATS2; Absinthe, China), and glyceraldehyde 3-phosphate dehydrogenase (GAPDH; Proteintech, United States). Horseradish peroxidase (HRP)-conjugated goat anti-rabbit IgG and anti-mouse IgG (Proteintech, United States) were used as secondary antibodies. Detection was performed using Chemiluminescent HRP Substrate (Merck Millipore, Germany), and signals were captured and observed using Molecular Imager^®^ ChemiDoc^TM^ XRS + (Bio-Rad, United States).

### Temporomandibular Joint Osteoarthritis Model Establishment

By using chemical method modeling-collagenase injection, a TMJOA animal model was established. The posterior pole of the TMJ condyle can be found behind the outer canthus of 12- to 18-week-old New Zealand rabbits. In its mouth opening position, with left forefinger tip pressing on the area of joint space, the syringe needle held in the right hand was thrust into the joint space inward, forward, and downward, parallel to the infraorbital margin. If the needle entered approximately 0.5 cm and was withdrawn without blood, approximately 0.25 ml of 4 mg/ml collagenase II (Yeasen, China) was injected into the TMJ, and this group was named OA group (the control group was injected with approximately 0.25 ml normal saline). 4 weeks later, the TMJOA rabbit model was identified by morphological evaluation and histological and molecular biological examination. All protocol and procedures employed *in vivo* were ethically reviewed and approved by the Institutional Animal Care and Use Committee at Zhejiang Laboratory Animal Center (Approval No. ZJCLA-IACUC-20050012) for the rational care and use of laboratory animals.

Under sterile conditions, the condyles of 12- to 18-week-old New Zealand rabbits were excised, and surrounding soft tissues were removed. Cartilage tissues on the condyles were separated and cut into 1-mm^3^ size; and after rinsing with PBS, 0.25% trypsin was added and treated for 30 min. Then, high-glucose DMEM containing 10% FBS was added to terminate dissociation. After discarding supernatant, 2 ml 0.3% collagenase (Yeasen, China) and 4 ml high-glucose DMEM containing 10% FBS were added and placed in 37°C. After 12 h, the MCCs were collected and cultured in six-well plates. When MCCs grew to 80–90% confluency, 10 ng/ml recombinant human interleukin-1 beta (IL-1β; Novoprotein, China) was added into the MCC medium and cultured in 37°C for 24 h, and then identification of TMJOA cell model, such as expression of OA-related factors, was launched.

### Small Extracellular Vesicle Treatment of Temporomandibular Joint Osteoarthritis Model

*In vivo*, normal and TMJOA model rabbits were randomly assigned into control groups and experimental groups, separately, and there were 6–8 TMJs of rabbits (i.e., 3–4 rabbits) in each group (one rabbit died in the OA group, which means that there were six TMJs of rabbits in that OA group), half male and half female. In the first *in vivo* experiment, the animals were divided into four groups: control group (rabbits in the former control group treated with PBS), OA group (rabbits in the former OA group treated with PBS), sEV group (rabbits in the former control group treated with sEVs), and OA^sEV^ group (rabbits in the former OA group treated with sEVs). This experiment was launched three times, for 4-week, 6–week, and 8-week observation, separately. In the second *in vivo* experiment, the animals were divided into four groups: OA group (rabbits in the former OA group treated with PBS), OA + sEV group (rabbits in the former OA group treated with sEV), OA + sEV^–/–*ATX*^ group (rabbits in the former OA group treated with sEV^–/–*ATX*^), and OA + sEV^*Ziritaxestat*^ group (rabbits in the former OA group treated with sEV^*Ziritaxestat*^). This experiment was launched three times, for 4 weeks, 6 weeks, and 8 weeks of observation, separately. In each *in vivo* experiment, approximately 200 μl (4–8) × 10^8^ BMSC-sEVs extracted from about 5.5 × 10^7^ BMSCs were injected into each TMJ in the experimental groups, once per side *per capita*. *In vitro*, approximately (2–4) × 10^8^ BMSC-sEVs aforementioned were used in the experimental groups (the control group was added with normal saline).

### Micro-Computed Tomography

Condyle specimens were scanned and analyzed by the high-resolution micro-computed tomography (micro-CT) scanner U-CT-XUHR (Milabs, Netherlands) and Imalytics Preclinical 2.1 software. Scanning parameters were 0.24 μA current, 50 kV voltage, 15-ms exposure time, and 30 deg/s angle speed. Subchondral cancellous bone was defined as the cancellous bone region 0.5 mm beneath the calcified cartilage–bone junction. The cylinder with volume of (π × 1.5^2^ × 1) mm^3^ was selected for each specimen to calculate the parameters, including bone volume fraction (BVF, BV/TV), bone surface/bone volume ratio (BS/BV), bone mineral density (BMD), trabecular thickness (Tb.Th), and trabecular spacing (Tb.Sp).

### Histology and Immunohistochemistry

Hematoxylin–eosin (HE) staining and specific staining for condylar cartilage (such as safranine O/fast green and Alcian blue) were adopted to identify the successful establishment of the TMJOA animal model.

Immunohistochemistry (IHC) for PCNA, Col-I, Col-II, ACAN, SOX9, MMP13, and RUNX2 was adopted to analyze the changes in the cartilage tissue of the condyle in the normal and TMJOA states with or without BMSC-sEV treatment.

### Scoring of Histology

The histologic observations of normal and TMJOA animal tissues were scored using the International Cartilage Regeneration & Joint Preservation Society (ICRS) Visual Histological Assessment Scale (Supplementary Table 1) and Wakitani Histological Grading Scale (Supplementary Table 2). The ICRS Visual Histological Assessment Scale contains six features (surface, matrix, cell distribution, cell population viability, subchondral bone, and cartilage mineralization), scored from 0 to 3, and the Wakitani Histological Grading Scale contains five categories (cell morphology, matrix staining, surface regularity, thickness of cartilage, and integration of donor with host adjacent cartilage), scored from 4 to 0, total maximum 14. Three independent blinded technicians from the Department of Pathology in our hospital were recruited to score, respectively.

### Laser Scanning Confocal Microscopy

Stem cell-derived small extracellular vesicles were dyed with Exo-Glow Exosome Labeling Kit (SBI, Japan). Single-stranded RNAs in the sEVs were fluorescently labeled in red by acridine orange (AO). F-actin was stained green with Actin-stain 488 fluorescent phalloidin (Cytoskeleton Inc., United States). Finally, the cell nucleus was stained blue with 4′,6-diamidino-2-phenylindole (DAPI; Thermo Fisher Scientific, United States). Fluorescence images were acquired on a TCS SP2 laser scanning confocal microscope (Leica Microsystems, Germany).

### Proliferation Assay

Cell proliferation of the MCCs was determined by the Cell Counting Kit-8 (CCK-8; Yeasen, China). MCCs were seeded in a 96-well plate in triplicate. They were treated with BMSC-sEVs for 24 h. During the next few days, the proliferation of the MCCs was detected at a wavelength of 450 nm, as measured by SpectraMax i3 (Molecular Devices, United States).

### Migration Assay

MCCs were cultured at 37°C until they were in the logarithmic phase and then treated with DMEM without FBS containing BMSC-sEVs for 24 h. The medium with the cells was then placed in the top chamber of a Transwell (Corning, United States) inserted into a 24-well plate. DMEM with 10% FBS was added to the bottom chamber, and the cells were cultured for approximately 24 h. Then, the cells that had migrated into the bottom chamber were fixed with 4% paraformaldehyde, dyed with 0.1% crystal violet, and observed by microscopy (Olympus, Japan).

### Cell Cycle Distribution Assay

The cell cycle distribution of the MCCs was determined by a Cell Cycle and Apoptosis Analysis Kit (Yeasen, China). Cell sediments were washed with PBS and resuspended in binding buffer. Propidium iodide (PI) was added to the solution and incubated for 15 min at room temperature in the dark. Then, the samples were analyzed by flow cytometry (FCM; CytoFLEX LX, Beckman, United States).

### Real-Time Quantitative PCR

Total RNA of the BMSCs and MCCs was extracted by TRIeasy^TM^ Total RNA Extraction Reagent (Yeasen, China) and reverse transcribed into cDNA using Hifair^®^ II 1st Strand cDNA Synthesis SuperMix for qPCR (Yeasen, China) and a Mastercycler instrument (Eppendorf, Germany). All qPCR programs were performed using Hieff UNICON^®^ Power qPCR SYBR Green Master Mix (Yeasen, China) and the CFX384^^TM^ Real-Time System (Bio-Rad, United States). mRNA expression was quantified by quantitative PCR (qPCR) using the 2^–ΔΔCT^ relative quantitation method, and GAPDH served as the internal control. The primers are listed in Table 1.

**TABLE 1:** The primer sequences of target genes.

Primer	Sequence (5′–3′)
PCNA-F	GCTCCATCCTGAAGAAGGTGCTG
PCNA-R	CGTGGGACGAGTCCATGCTTTG
COL2A1-F	GTCCTGTGCGACGACATAATCT
COL2A1-R	GGCAGTGGCGAGGTCAGTAG
ACAN-F	GCTACGACGCCATCTGCTACAC
ACAN-R	GTCCTCCTCACCGCCCACTC
SOX9-F	GAAGCTCTGGAGACTGCTGAA
SOX9-R	CCCATTCTTCACCGACTTCCT
MMP13-F	TCCAGTTTGCAGAGAGCTACC
MMP13-R	GACTGCATTTCTCGGAGCCT
RUNX2-F	GAACCCAGAAGGCACAGACAGAAG
RUNX2-R	GAGGCGGGACACCTACTCTCATAC
ATX-F	TGTCCTCCTTCATCCTGCCTCAC
ATX-R	TGTTCAATGTCACGCACCCTAGC
RHOA-F	ATTGTCGGTGATGGTGCTTGTGG
RHOA-R	TGGGGACATACACCTCTGGGAAC
LATS1-F	GTGACCATCCACGGCAAGATAGC
LATS1-R	GTGCTAGACATCGCTGGTGCTG
LATS2-F	CCAACTCCTTCAACAGCCAGCAG
LATS2-R	CAGCACCCGCACACTCTTCAC
AKT2-F	GGTCGCCAACAGCCTCAAGC
AKT2-R	ACCGCCACTTCCATCTCCTCAG
AKT3-F	ACAGATGCAGCCACCATGAAGAC
AKT3-R	GAACGGCAACCTCCCACACATC
GAPDH-F	AGGTCGGAGTGAACGGATTT
GAPDH-R	GATCTCGCTCCTGGAAGATGG

*PCNA, proliferating cell nuclear antigen; COL2A1, type II collagen; ACAN, aggrecan; SOX9, SRY-related high-mobility group box 9; MMP13, matrix metalloproteinase 13; RUNX2, RUNX family transcription factor 2; ATX, autotaxin; RhoA, ras homolog family member; ALATS 1/2, large tumor suppressor kinase 1/2; GAPDH, glyceraldehyde 3-phosphate dehydrogenase.*

### Knockdown and Inhibition of Autotaxin

To knock down the expression of autotaxin, three gene-specific short interfering RNAs (siRNAs) (Table 2) were synthesized (GenePharma, China) and used. The cells were transfected with these siRNAs using Lipofectamine RNAiMAX Transfection Reagent (Thermo Fisher Scientific, United States). After 48 h, the knockdown efficacy was confirmed by qPCR and immunoblotting to quantitate the expression of the genes at both the transcription and translation levels in the cells and in their sEVs. To inhibit the expression of autotaxin, a specific inhibitor, ziritaxestat (GLPG1690) (Selleck, United States), was used.

**TABLE 2:** The siRNA sequences targeting the autotaxin gene.

siRNA		Sequence (5′–3′)
Negative control	Sense	UUCUCCGAACGUGUCACGUTT
	Anti-sense	ACGUGACACGUUCGGAGAATT
siAutotaxin-1	Sense	GCAGCAAAGUCAUGCCUAATT
	Anti-sense	UUAGGCAUGACUUUGCUGCTT
siAutotaxin-2	Sense	GCAGUGCUUUAUCGGACUATT
	Anti-sense	UAGUCCGAUAAAGCACUGCTT
siAutotaxin-3	Sense	GGUCUGGAAUUAUUUCCAATT
	Anti-sense	UUGGAAAUAAUUCCAGACCTT

### Statistical Analysis

Statistical analyses were performed with SPSS 19.0 (IBM Corp., United States), and the figures were made with GraphPad Prism 8 software (GraphPad Software, United States). Both parametric and non-parametric inferential statistics were used depending on whether the data were normally distributed tested by Kolmogorov–Smirnov test. Two-tailed *t*-test and Mann–Whitney *U* test were used to identify differences between groups. One-way analysis of variance (ANOVA) and Kruskal–Wallis tests were carried out for multiple group comparisons. Three replicates were set for each treatment. The data are presented as the mean ± standard error of the mean (SEM). *P* value less than 0.05 was considered statistically significant.

## Results

### Acquisition and Identification of the Bone Marrow Mesenchymal Stem Cell-Derived Small Extracellular Vesicles

**FIGURE 1:**
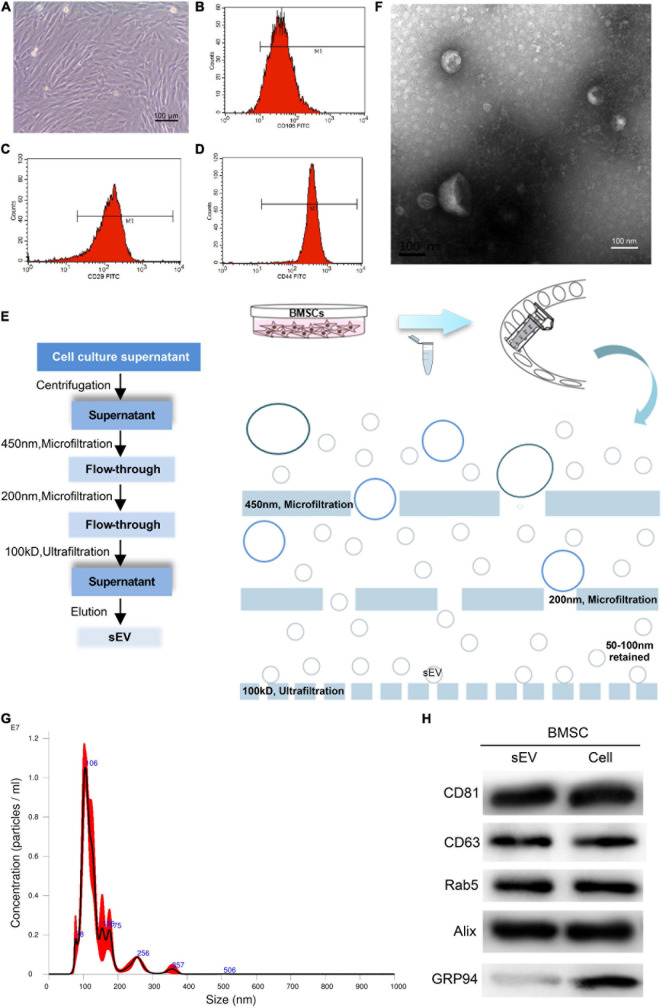
Acquisition and identification of bone marrow mesenchymal stem cell-derived small extracellular vesicles (BMSC-sEVs). **(A)** Morphologic observation of BMSCs under an optical microscope (100×). **(B–D)** Identification of BMSCs by CD105, CD29, and CD44 using flow cytometry (FCM). **(E)** Flow and schematic diagram of sEV isolation and purification. **(F)** Electron microscopy analysis of BMSC-sEVs. **(G)** Nanoparticle tracking analysis of BMSC-sEVs. **(H)** Immunoblotting analysis of exosomal positive and negative markers in BMSC-sEVs (left) and in donor whole-cell lysates (right).

Human BMSCs were harvested from the healthy human iliac bone marrow. Viewed by optical microscopy, their cell form was fibroblast-like, with a long barracuda, fish, vortex, or reticular arrangement (Figure 1A). By FCM, the BMSC markers CD105, CD29, and CD44 were identified (whose counts were 96.1, 98.7, and 99.4%, respectively), and the results complied with the BMSC identification criteria (Figures 1B–D).

By centrifugation, microfiltration (0.45 and 0.20 μm) and ultrafiltration (100 kD), BMSC-sEVs were obtained (Figure 1E). Through transmission electron microscopy (TEM), many “cup-shaped” and “tea saucer-shaped” vesicles with membrane structures could be seen, with diameters of approximately 30–120 nm. The size and morphology of the BMSC-sEV samples observed under the microscope conformed to the relevant definitions and characteristics of sEVs (Figure 1F). The NTA results showed that the particle size distribution of the BMSC-sEV samples obtained in this experiment was concentrated at approximately 106 nm. Each 1 ml BMSC-sEV sample contained approximately 1.05 × 10^7^ particles, derived from approximately 5.5 × 10^7^ BMSCs (Figure 1G). The NTA results suggested that the BMSC-sEV samples obtained in this experiment conformed to the relevant definitions and characteristics of sEVs, consistent with the TEM results mentioned above. In addition, the results showed that the concentration and purity of the sEV samples from the BMSC sources were satisfactory.

sEVs can also be identified by positive and negative markers. Positive markers of sEVs, such as CD63, CD81, Rab5, and Alix, were clearly detected in the BMSC-sEVs and their donor cells, whereas the negative marker GRP94 was only detected in the BMSCs (Figure 1H).

These results show that the sEV separation and purification technology used in this study is reliable and effective; the BMSC-sEV samples are in line with the recognized definition and identification standards of sEVs, with satisfactory concentration and purity.

### Bone Marrow Mesenchymal Stem Cell-Derived Small Extracellular Vesicles Contributed to Temporomandibular Joint Osteoarthritis Reconstruction *in vivo*

#### Histologic Assessment of Temporomandibular Joint Osteoarthritis Condylar Cartilage Treated With Bone Marrow Mesenchymal Stem Cell-Derived Small Extracellular Vesicles

**FIGURE 2:**
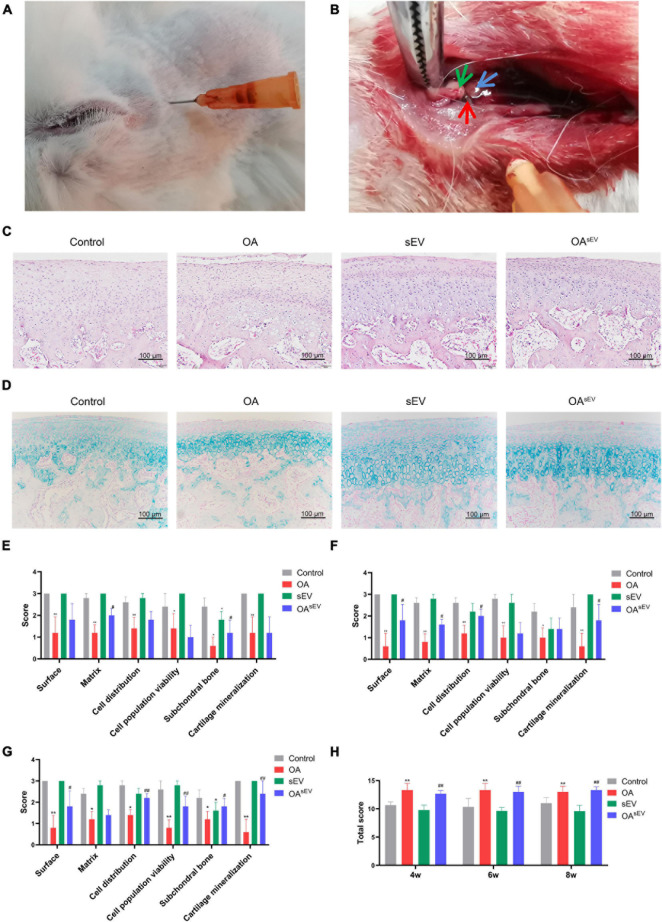
Histologic assessment of temporomandibular joint osteoarthritis (TMJOA) condylar cartilage treated with bone marrow mesenchymal stem cell-derived small extracellular vesicles (BMSC-sEVs). **(A)** Needle insertion site to establish the rabbit TMJOA model using the chemical injection method. **(B)** Anatomy of the establishment of the TMJOA model by the chemical injection method (green arrow pointing to the TMJ disc, blue arrow pointing to the condylar cartilage, and red arrow pointing to the needle tip). **(C)** Hematoxylin–eosin (HE) staining observation of the establishment of the TMJOA model (200×). **(D)** Alcian blue staining observation of the establishment of the TMJOA model (200×). **(E–G)** Histologic scoring of the condyle of normal and the TMJOA model group (treated with BMSC-sEVs for 4, 6, and 8 weeks, respectively) by the ICRS Grading Scale. **(H)** Histologic scoring of the condyle of the normal and TMJOA model groups by the Wakitani Grading Scale. *: Compared with the control group, #: Compared with the OA group. */#*P* < 0.05, **/##*P* < 0.01.

The rabbit TMJOA model was established according to the method described above (Figure 2A). During the operation, the injection point and depth were accurate; the needle entered the supra-fissure of the TMJ capsule between the condyle and the joint disc (Figure 2B). Gross observation results showed that the condyles in OA group relatively lacked integrity (Supplementary Figure 1A). Micro-CT results suggested that BVF, BMD, and Tb.Th were significantly reduced and that BS/BV and Tb.Sp were increased in the OA group (Supplementary Figures 1B–G). The results of HE and Alcian blue staining for cartilage showed that each layer of the OA group’s cartilage (fiber layer, proliferation layer, mature cartilage layer, and calcified cartilage layer) was thin compared to the control group, and the cartilage structure and level were not well organized (Figures 2C,D). The above results showed that the rabbit TMJOA model was successfully established.

The histological scores of the OA group were worse than those of the control group. Better histological scores were not observed in the sEV group than in the control group. Compared with the OA group, however, the histology scores of the OA^sEV^ group were better (Figures 2E–H). These results suggested that BMSC-sEVs could induce histologic repair of TMJOA condylar cartilage.

#### Expression of Osteoarthritis-Related Factors in Temporomandibular Joint Osteoarthritis Condylar Cartilage Treated With Bone Marrow Mesenchymal Stem Cell-Derived Small Extracellular Vesicles

**FIGURE 3:**
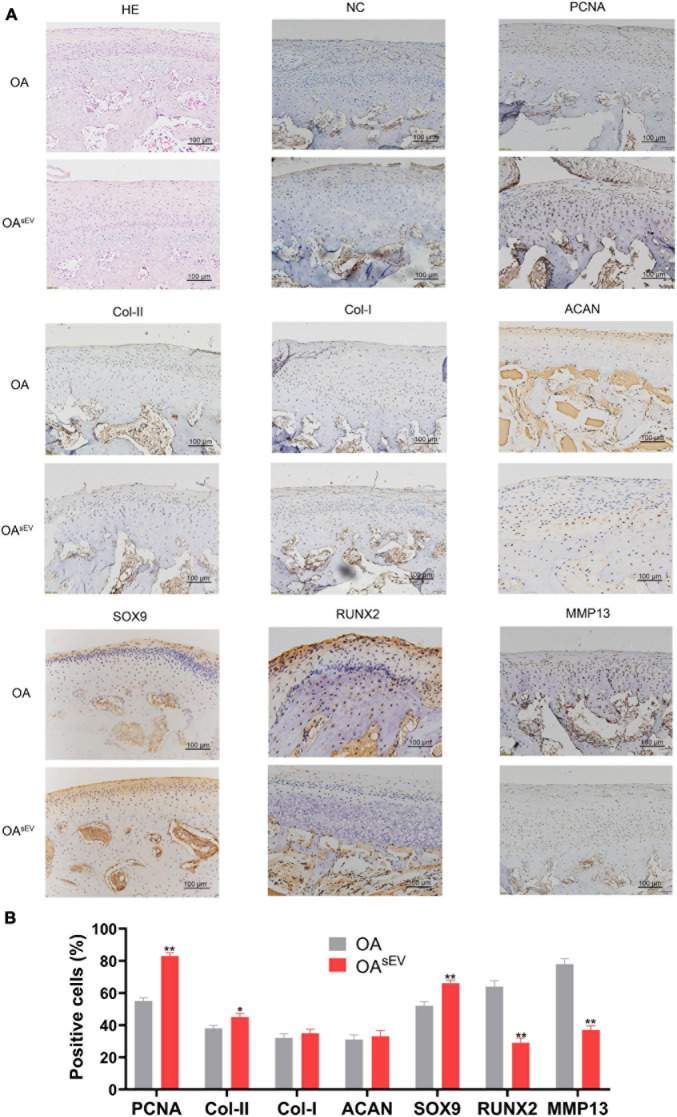
Histologic expression of osteoarthritis (OA)-related factors in temporomandibular joint osteoarthritis (TMJOA) condylar cartilage treated with bone marrow mesenchymal stem cell-derived small extracellular vesicles (BMSC-sEVs). **(A)** Hematoxylin–eosin (HE) and immunohistochemistry (IHC) analysis of the expression of proliferating cell nuclear antigen (PCNA), cartilage-forming factors, and cartilage inflammation-related factors in the OA control group and the OA treated with BMSC-sEV group (OA^sEV^) (200×). NC, negative control. **(B)** Semiquantitative analysis of IHC. *P < 0.05, **P < 0.01.

The HE staining results showed that compared with the untreated OA group, the condylar tissue of the OA^sEV^ exhibited an increase in cartilage lacuna. The cartilage cell proliferation was obvious. Hypertrophic cartilage cells (with larger cartilage lacuna) could also be seen in the deep area of the bone under the cartilage, which suggested more obvious hyaline cartilage formation (Figure 3A). The IHC results showed that compared with the untreated OA group, the condylar tissue of the OA^sEV^ exhibited significantly upregulated expression of cell proliferation-related factor-PCNA, Col-II, ACAN, SOX9 (with no significant difference in the expression of Col-I), and the downregulated expression of cartilage inflammation-related factor-MMP13 and RUNX2 (Figures 3A,B).

Combined with the above results, the *in vivo* experiments confirmed that BMSC-sEVs played an important role in promoting cartilage reconstruction in TMJOA.

### Bone Marrow Mesenchymal Stem Cell-Derived Small Extracellular Vesicles Upregulated Cartilage Cell Activity and Cartilage Reconstruction Factor Expression in Temporomandibular Joint Osteoarthritis *in vitro*

#### Acquisition and Identification of Mandibular Condylar Cartilage Cells

**FIGURE 4:**
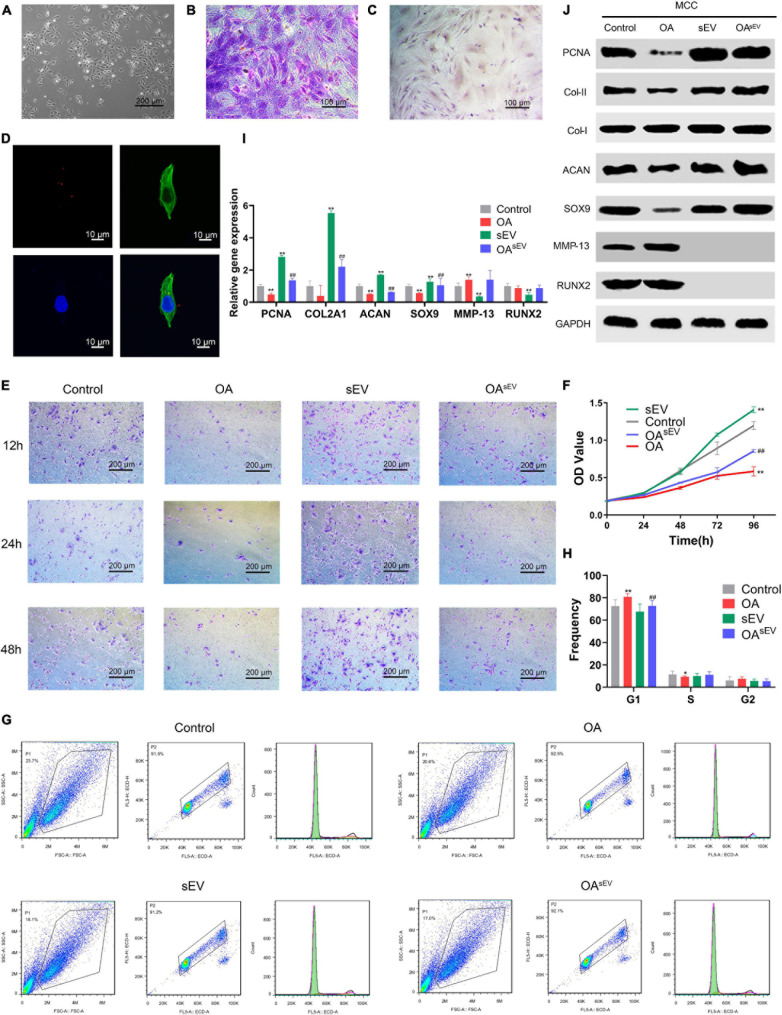
Bone marrow mesenchymal stem cell-derived small extracellular vesicles (BMSC-sEVs) promoted cartilage reconstruction of temporomandibular joint osteoarthritis (TMJOA) *in vitro*. **(A)** Morphologic observation of mandibular condylar chondrocytes (MCCs) under an optical microscope (100×). **(B)** Identification of MCCs using toluene blue staining (200×). **(C)** Immunohistochemistry (IHC) identification of MCCs targeting type II collagen (Col-II) (200×). **(D)** Laser scanning confocal microscopy analysis of BMSC-sEV (red) internalization by MCCs. **(E)** Analysis of the cell migration activity of MCCs by Transwell assays at 12, 24, and 48 h (100×). **(F)** Analysis of the cell proliferation activity of MCCs by Cell Counting Kit-8 (CCK-8). **(G,H)** Detection of the cell cycle distribution of MCCs by flow cytometry (FCM) and statistical analysis. **(I,J)** The expression of PCNA, cartilage-forming factors, and cartilage inflammation-related factors at the transcriptional and translational levels by quantitative polymerase chain reaction (qPCR) and immunoblotting, respectively. *: Compared with the control group, #: Compared with the OA group. */#*P* < 0.05, **/##*P* < 0.01.

MCCs were extracted from healthy New Zealand rabbit TMJ condyles and identified by specific staining and immunocytochemistry (ICC). The results showed that the MCCs were irregularly distributed in the polygon or strip, similar to short fibroblasts, and the MCCs stained with toluene blue (TB) and Col-II using the ICC technique met the identification criteria (Figures 4A–C).

#### Bone Marrow Mesenchymal Stem Cell-Derived Small Extracellular Vesicles Could Be Internalized by Mandibular Condylar Chondrocytes

Then, BMSC-sEVs were analyzed using fluorescent dyes and laser scanning confocal microscopy (Figure 4D). BMSC-sEVs harboring red dye-labeled RNAs were internalized in the MCCs, which showed that BMSC-sEVs could be internalized by MCCs.

#### Enhancement of the Biological Activity of Mandibular Condylar Chondrocytes Treated With Bone Marrow Mesenchymal Stem Cell-Derived Small Extracellular Vesicles

The migration test using Transwell assays showed that compared with the control group, the migratory ability of the MCCs in the OA group (established by the aforementioned IL-1β) was weakened; in contrast, the migration of the MCCs in the sEV group was enhanced. Furthermore, the migratory ability of the MCCs in the OA^sEV^ group was increased compared with that in the OA group. In addition, the treatment duration (12, 24, and 48 h) did not change the results (Figure 4E).

The proliferation of TMJOA MCCs was enhanced by BMSC-sEVs compared with their corresponding control group (Figure 4F). In addition, the FCM results showed that compared with the control group, the cell cycle of the MCCs in the OA group was stagnated in the G1 stage relative to the control group; in contrast, stagnation of the cell cycle of the MCCs in the OA^sEV^ group was alleviated to some degree (Figures 4G,H).

#### Expression of the Relevant Factors of Mandibular Condylar Chondrocyte Cartilage Reconstruction Treated With Bone Marrow Mesenchymal Stem Cell-Derived Small Extracellular Vesicles

Compared with the OA group, in the OA^sEV^ group, the expression of PCNA, Col-II, ACAN, and SOX9 was upregulated (Col-I expression did not show a significant difference), while the expression of RUNX2 and MMP13 was decreased, indicating that BMSC-sEVs could promote TMJOA cartilage reconstruction (Figures 4I,J).

Combining the above results, the *in vitro* experiments confirmed that BMSC-sEVs significantly promoted cartilage reconstruction in TMJOA.

### The Mechanism of the Effect of Bone Marrow Mesenchymal Stem Cell-Derived Small Extracellular Vesicles on the Acceleration of Cartilage Reconstruction in Temporomandibular Joint Osteoarthritis

#### The Important Role of the Autotaxin and Hippo Pathways in Temporomandibular Joint Osteoarthritis Using Bioinformatic Analysis

Based on the Gene Expression Omnibus (GEO) database, the gene expression chips of normal humans and OA patients, GSE6119, GSE19664, GSE27357, etc., were obtained. Using GEO2R^1^, KOBAS 3.0^2^, DAVID 6.8^3^ and other analysis methods, functional Gene Ontology (GO) analysis and Kyoto Encyclopedia of Genes and Genomes (KEGG) signaling pathway analysis were adopted to determine the differentially expressed factors and signaling pathways involved in TMJOA.

Take the GSE6119 data in the GEO database as an example. Among the differentially expressed genes, many Hippo pathway-related factors, such as Bmp7, Bmp6, Tgfb2, Bmp4, Bmp2, Ccnd1, Id1, Tgfb3, Wnt9a, Fgf1, and Dlg4, were present. In addition, the Hippo pathway was ranked at the top 1% among the large number of signaling pathways involved. These results suggested that the Hippo pathway was differentially expressed between OA tissue and healthy tissue and played a more important role than the other signaling pathways in TMJOA.

#### Upregulation of Key Factors of the Hippo Pathway in Mandibular Condylar Chondrocytes Treated With Bone Marrow Mesenchymal Stem Cell-Derived Small Extracellular Vesicles

**FIGURE 5:**
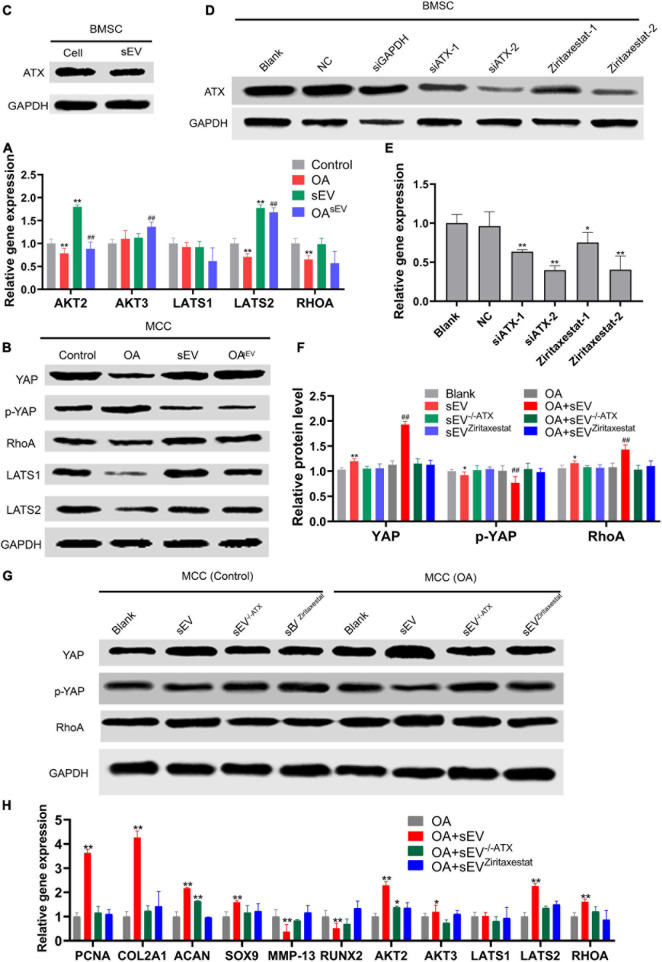
Effect of autotaxin on the mechanism by which bone marrow mesenchymal stem cell-derived small extracellular vesicles (BMSC-sEVs) accelerated cartilage reconstruction in temporomandibular joint osteoarthritis (TMJOA). **(A,B)** Expression of key factors [such as Yes-associated protein (YAP), ras homolog family member (RhoA), etc.] of the Hippo pathway in mandibular condylar chondrocytes (MCCs) at the transcriptional and translational levels by quantitative polymerase chain reaction (qPCR) and immunoblotting (IB), respectively. **(C)** Expression of autotaxin in BMSCs and their secreted sEVs by immunoblotting. **(D,E)** Efficiency of knockdown and inhibition of BMSCs targeting autotaxin at the translation and transcription levels by immunoblotting and qPCR, respectively (NC, negative control; siGAPDH, positive control; siATX-1/2, two siRNAs targeting autotaxin; ziritaxestat-1/2, autotaxin inhibitors at concentrations of 2 and 10 μM, respectively). *P < 0.05, **P < 0.01. **(F,G)** Expression of key factors (such as YAP, RhoA, etc.) of the Hippo pathway in normal MCCs or those with autotaxin knockdown or inhibition at the translation level by immunoblotting and statistical analysis. **(H)** Expression of key factors (such as YAP, RhoA, etc.) of Hippo pathway in normal MCCs and those with autotaxin knockdown or inhibition at the transcription level by qPCR. *: Compared with the control group, #: Compared with the OA group. */#*P* < 0.05, **/##*P* < 0.01.

Compared with the untreated OA group, MCCs in the OA^sEV^ group had upregulated expression of YAP, key factors of the Hippo pathway, and its upstream molecules, such as ras homolog family member A (RhoA), and downstream molecules, such as LATS 1/2. Conversely, the expression trend of p-YAP (S127) was contrary to the above factors, indicating that the BMSC-sEVs inhibited YAP phosphorylation in MCCs, thereby inhibiting the Hippo pathway, and played a role in promoting proliferation and cartilage regeneration in TMJOA (Figures 5A,B).

#### No Upregulated Expression of Key Factors of the Hippo Pathway in Mandibular Condylar Chondrocytes Treated With Bone Marrow Mesenchymal Stem Cell-Derived Small Extracellular Vesicles When Autotaxin Was Inhibited or Knocked Down

The IB results showed that BMSCs and their secreted sEVs expressed autotaxin (Figure 5C). In MCCs treated with BMSC-sEVs, in which autotaxin was inhibited or knocked down (Figures 5D,E), key factors of the Hippo pathway, such as RhoA and YAP, were no longer upregulated. These results preliminarily revealed that MSC-sEVs promoted cartilage reconstruction of TMJOA *via* the autotaxin–YAP signaling axis (Figures 5F–H).

#### No Cartilage Reconstruction of Mandibular Condylar Chondrocytes Treated With Bone Marrow Mesenchymal Stem Cell-Derived Small Extracellular Vesicles When Autotaxin Was Inhibited or Knocked Down

**FIGURE 6:**
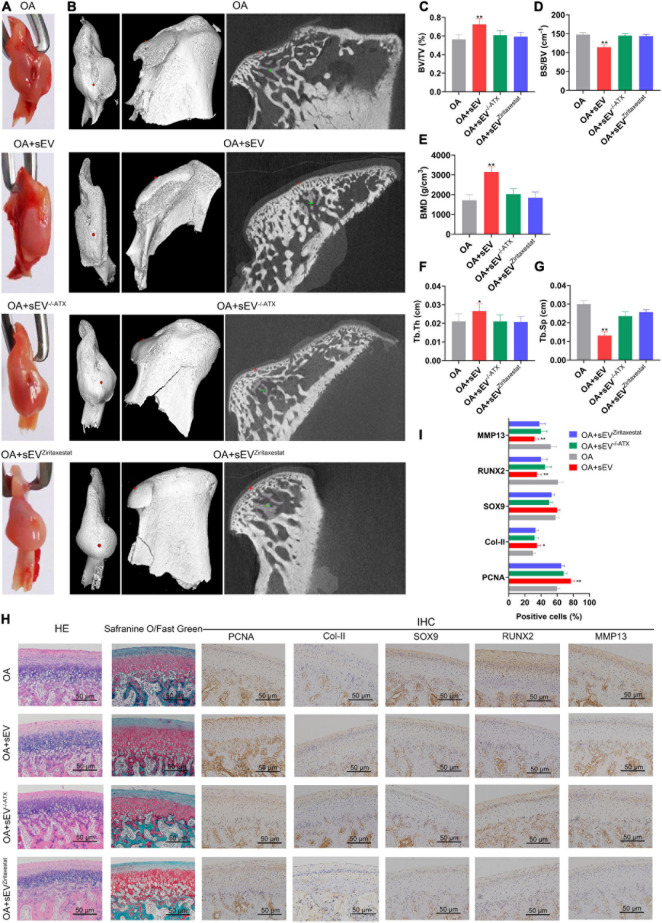
No cartilage reconstruction of mandibular condylar chondrocytes (MCCs) treated with bone marrow mesenchymal stem cell-derived small extracellular vesicles (BMSC-sEVs) was observed when autotaxin was inhibited or knocked down. **(A)** Gross observation of condyles of temporomandibular joint osteoarthritis (TMJOA) animal model treated with BMSC-sEVs, normal and autotaxin knockdown or inhibition. **(B)** Micro-CT images of condyles of TMJOA animal model treated with BMSC-sEVs, normal and autotaxin knockdown or inhibition. **(C–G)** Parameters of micro-CT analysis of these groups, including bone volume fraction (BVF), bone surface/bone volume ratio (BS/BV), bone mineral density (BMD), trabecular thickness (Tb.Th), and trabecular spacing (Tb.Sp). **(H)** Hematoxylin–eosin (HE), safranin O/fast green, and immunohistochemistry (IHC) analysis of condyles of TMJOA animal model treated with BMSC-sEVs, normal and autotaxin knockdown or inhibition for 4 weeks (200×). **(I)** Semiquantitative analysis of IHC. *P < 0.05, **P < 0.01.

Gross observation results showed that compared with the other OA groups, the OA^sEV^ group had better integrity of condyles (Figure 6A). Micro-CT results suggested that BVF, BMD, and Tb.Th were significantly increased in the OA^sEV^ group and that BS/BV and Tb.Sp were reduced in this group (Figures 6C–G). These data proved that damage in the surface of condyle cartilage and subchondral bone in TMJOA could be reversed by sEV-autotaxin derived from BMSCs (Figures 6C–G).

HE and safranin O/fast green staining results showed that compared with the untreated OA group, the condylar tissue of the OA^sEV^ group exhibited an increase in cartilage lacuna (Figure 6), which is consistent with the results above (Figure 3A). In contrast, the condylar tissue of the OA^sEV^, in which autotaxin had been knocked down or inhibited (OA + sEV^–/–*ATX*^, OA + sEV^*Ziritaxestat*^), no longer exhibited an increase in cartilage lacuna (Figure 6). The IHC results showed that compared with the untreated OA group, the condylar tissue of the OA^sEV^ group showed upregulated expression of PCNA, Col-II, and SOX9 and downregulated the expression of RUNX2 and MMP13 (Figures 6H,I and Supplementary Figure 2), which is consistent with the results above (Figures 3A,B). In contrast, the condylar tissue of the OA^sEV^, in which autotaxin had been knocked down or inhibited (OA + sEV^–/–*ATX*^, OA + sEV^Ziritaxestat^), did not show changes in the expression of these factors (Figures 6H,I and Supplementary Figure 2).

## Discussion

BMSCs have been widely used in many occasions, such as defect repairing and tissue regeneration, which are based on several experimental and clinical studies. A recent study reported that BMSC implantation appeared to be an effective and safe treatment for chondral defects at up to 10 years (Teo et al., 2019). Nevertheless, there are some problems with the treatment of TMJOA with MSCs, one of which is their inefficiency. To improve their transplantation and survival, some studies have placed MSCs into advanced carrier systems to improve their retention, vitality, growth, and differentiation. MSCs can be loaded into the material, such as by inoculation into a macroporous scaffold or into hydrogel (Armiento et al., 2018). Bio-hydrogels developed by the research team at our university, with fast light curing and long degradation cycles, can be loaded with BMSCs to promote cell migration, a nutritional supply, and cell survival.

Nevertheless, in clinical applications, the number of MSCs required to achieve the desired therapeutic results is still very large. This leads to another problem: forcing MSCs to multiply in large quantities to increase their yields can induce MSCs to show a tumorous tendency. In addition, there is unevenness in the expanded MSC population, which leads to less stable and reliable treatment by MSCs from different sources and batches, limiting further clinical applications of MSCs. Currently, it is widely proposed that MSCs may play their multiple biological roles by producing extracellular vesicles of varying sizes (Phinney and Pittenger, 2017). Considering this, using BMSC-sEVs instead could be a reliable and effective treatment for TMJOA, which is supported by the results of this study.

BMSC-sEVs can overcome problems arising from direct applications of MSCs, and in recent years, they have been found to have a therapeutic effect on OA. BMSC-sEVs can promote the repair of cartilage defects by promoting cell proliferation and infiltration, and they can also regulate cell proliferation, migration, and vascular formation through a variety of miRNAs (Qin et al., 2016; Cosenza et al., 2017; Zhang et al., 2018). When co-cultured with OA chondrocytes, BMSC-sEVs highly expressed cyclooxygenase-2 (COX-2) and pro-inflammatory interleukin, inhibited the activity of tumor necrosis factor (TNF)-alpha-induced collagen, and promoted the synthesis of ACAN and type II collagen (Vonk et al., 2018). In addition, embryo-derived sEVs could reduce matrix degradation and cartilage destruction in mice and promote cartilage regeneration (Guo et al., 2016; Wang et al., 2017). A recent study indicated that human BMSC-sEVs overexpressing miRNA-26a-5p could alleviate OA by reducing prostaglandin endoperoxide synthase 2 (PTGS2) (Jin et al., 2020).

However, the role of BMSC-sEVs in TMJOA is still unclear. The TMJ has two motor patterns, sliding and rotation with a disc inside, which means that its function and mechanism are complex. In addition, unlike other joints of the body derived from the mesoderm, the TMJ is derived from the ectoderm, which means that there are many differences in its histogenesis, inflammation, and immunity. Therefore, determining whether MSC-sEVs have a similar effect on TMJOA is necessary because relevant research is very limited. Recent studies have suggested that MSC-sEVs can promote cartilage repair in rats and reduce TMJOA-related pain (Zhang et al., 2019; Lee et al., 2020). The results of this article showed that human BMSC-sEVs could promote cartilage reconstruction in rabbit TMJOA and increase cell proliferation activity. Cartilage cell proliferation-, formation-, and matrix regeneration-related factors were all enhanced (Figures 3, 6).

Regarding the mechanism by which MSC-sEVs promote OA cartilage cell proliferation, migratory activity, and cartilage regeneration, some studies have pointed to the Hippo signaling pathway, which plays an important role in cell proliferation, tissue regeneration, and stem cell function. Based on the GEO database, gene chips from OA patients and a normal human population were obtained and analyzed. The Hippo signaling pathway was significantly differentially expressed in the OA tissues, and the role of the pathway was more important than the other signaling pathways involved. Previous studies have shown that synovial MSC-sEVs activate the key factor of the Hippo signaling pathway, YAP, which increases the proliferation and migratory activity of chondrocytes in an OA model in mice (Tao et al., 2017). In addition, YAP can also phosphorylate Akt and thus promote cell survival. After TMJOA rats were treated with MSC-sEVs, Akt expression was increased, which in turn promoted cartilage regeneration (Zhang et al., 2019).

Nevertheless, the role of YAP in cartilage regeneration is not clear. The YAP-TEA domain (TEAD) complex can increase cell proliferation potential by activating the SOX6 promoter. However, YAP can activate the Wnt/β-catenin pathway, reducing the ability of cells to differentiate into cartilage, and YAP also reduces the expression of the RUNX2-mediated COL10A1 gene, which hinders the maturation of chondrocytes (Li et al., 2017). Therefore, the role and mechanisms of the Hippo pathway in OA with YAP as the key factor are worth exploring in further detail. The results from this study showed that BMSC-sEVs can promote an increase in YAP expression in TMJOA chondrocytes and that the level of YAP phosphorylation is downregulated, indicating that the Hippo pathway is inhibited, and this promotes cell survival and proliferation (Figure 5). Nevertheless, further mass spectrometry and sequencing of proteins and miRNAs are required for further study of the role of the Hippo pathway and the relationships among other pathways in this mechanism.

The upstream factors of the Hippo signaling pathway have not been explained thoroughly. A recent study revealed the role of the RhoA-striatin-interacting phosphatase and kinase (STRIPAK) signaling axis in the Hippo pathway (Chen et al., 2019). After stimulation by serum or lysophosphatidic acid (LPA), active RhoA binds to STRIPAK, which induces the binding and dephosphorylation of mammalian STE20-like protein kinase (MST1/2) and mitogen-activated protein kinase kinase kinase kinases (MAP4Ks). This results in YAP dephosphorylation, Hippo pathway inhibition, and the expression of cell proliferation- and cartilage regeneration-related factors. Autotaxin plays a role in the process of wound healing, angiogenesis factor formation, chemotaxis and the cell cycle (Knowlden and Georas, 2014). Studies have shown that autotaxin promotes MSC migration and cytoskeletal rearrangement (Ryu and Han, 2015). It has also been found that autotaxin binds to sEVs, which in turn transport LPA to target cells (Jethwa et al., 2016).

The results of this study showed that the BMSC-sEVs could carry autotaxin and that sEV-autotaxin could induce condylar cartilage repair and upregulate the expression of the aforementioned key factors of the Hippo pathway (Figures 5, 6). Nevertheless, further study of the mechanism is still needed by using a knockout animal model and by analyzing the detailed interaction between autotaxin and the Hippo signaling pathway. Finally, clinical trials of BMSC-sEVs in TMJOA patients are expected in the near future.

## Conclusion

**FIGURE 7:**
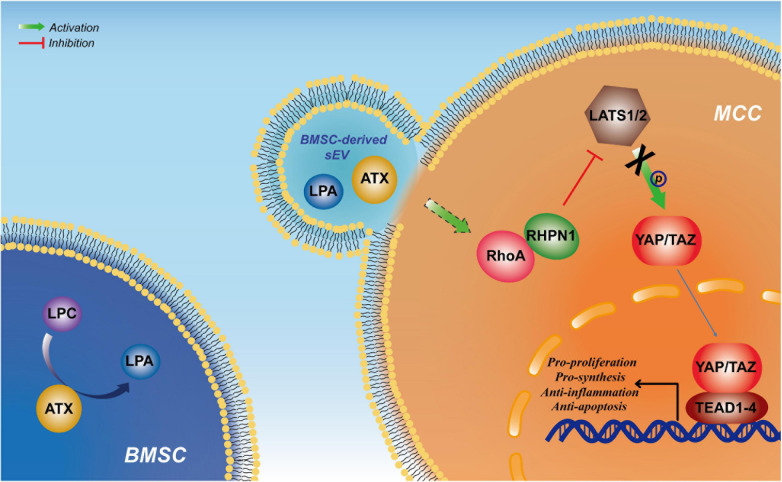
Schematic suggesting that bone marrow mesenchymal stem cell-derived small extracellular vesicles (BMSC-sEVs) induce cartilage reconstruction of temporomandibular joint osteoarthritis (TMJOA) *via* the autotaxin–Yes-associated protein (YAP) signaling axis.

Our study reveals that BMSC-derived sEVs could induce cartilage reconstruction in TMJOA *via* the autotaxin–YAP signaling axis (Figure 7), which could be expected to play an important role in the clinical application of sEVs in TMJOA and increase comprehension of the underlying mechanism.

## Data Availability Statement

The original contributions presented in the study are included in the article/Supplementary Material, further inquiries can be directed to the corresponding author/s.

## Ethics Statement

The animal study was reviewed and approved by Institutional Animal Care and Use Committee at Laboratory Animal Center of Zhejiang University. Written informed consent was obtained from the owners for the participation of their animals in this study.

## Author Contributions

YW and MW conceived and designed the research. YW and MZ performed the main experiments and wrote the manuscript. WL performed the data statistics and interpretation. YY and RM involved in data validation. ZZ and MW reviewed and revised the manuscript. All authors read and approved the final manuscript.

## Conflict of Interest

The authors declare that the research was conducted in the absence of any commercial or financial relationships that could be construed as a potential conflict of interest.

## Footnotes

1 http://www.ncbi.nlm.nih.gov/geo/geo2r

2 http://kobas.cbi.pku.edu.cn/kobas3

3 https://david.ncifcrf.gov

## Supplementary Material

Supplementary Figure 1 Gross observation and Micro-CT of condyles of TMJOA animal model. **(A)** Gross observation of condyles of TMJOA animal model. **(B)** Micro-CT images of condyles of TMJOA animal model. **(C–G)**. Parameters of micro-CT analysis of TMJOA animal model, including BVF, BS/BV, BMD, Tb.Th and Tb.Sp. ^∗^*P* < 0.05, ^∗∗^*P* < 0.01.

Supplementary Figure 2 Histologic and IHC analysis of condyles of TMJOA model treated with multiple kinds of BMSC-sEVs. HE, safranin O/fast green and IHC analysis of condyles of TMJOA animal model treated with BMSC-sEVs, normal and autotaxin knockdown or inhibition for 6 w and 8 w, respectively (200×).

## Abbreviations

TMJOA: temporomandibular joint osteoarthritis
BMSC-sEVs: bone marrow mesenchymal stem cell-derived small extracellular vesicles
ATX: autotaxin
YAP: Yes-associated protein
PCNA: proliferating cell nuclear antigen
Col-II/I: type II/I collagen
ACAN: aggrecan
SOX9: SRY-related high-mobility group box 9
MMP13: matrix metalloproteinase 13
RUNX2: RUNX family transcription factor 2
RhoA: ras homolog family member A
IB: immunoblotting
IHC: immunohistochemistry
qPCR: quantitative polymerase chain reaction.

## References

[B1] Abouelhuda A. M. Khalifa A. K. Kim Y. K. Hegazy S. A. (2018). Non-invasive different modalities of treatment for temporomandibular disorders: review of literature. *Korean Assoc. Oral Maxillofac. Surg.* 44 43–51. 10.5125/jkaoms.2018.44.2.43 29732308 PMC5932270

[B2] Armiento A. R. Stoddart M. J. Alini M. Eglin D. (2018). Biomaterials for articular cartilage tissue engineering: learning from biology. *Acta Biomater.* 65 1–20. 10.1016/j.actbio.2017.11.021 29128537

[B3] Chen K. Man C. Zhang B. Hu J. Zhu S. S. (2013). Effect of in vitro chondrogenic differentiation of autologous mesenchymal stem cells on cartilage and subchondral cancellous bone repair in osteoarthritis of temporomandibular joint. *Int. J Oral Maxillofac. Surg.* 42 240–248. 10.1016/j.ijom.2012.05.030 22763137

[B4] Chen R. Xie R. Meng Z. Ma S. Guan K. L. (2019). STRIPAK integrates upstream signals to initiate the Hippo kinase cascade. *Nat. Cell Biol.* 21 1565–1577. 10.1038/s41556-019-0426-y 31792377

[B5] Cosenza S. Ruiz M. Toupet K. Jorgensen C. Noël D. (2017). Mesenchymal stem cells derived exosomes and microparticles protect cartilage and bone from degradation in osteoarthritis. *Sci. Rep.* 7:16214. 10.1038/s41598-017-15376-8 PMC5701135 29176667

[B6] García-García A. de Castillejo C. L. Méndez-Ferrer S. (2015). BMSCs and hematopoiesis. *Immunol. Lett.* 168 129–135. 10.1016/j.imlet.2015.06.020 26192443

[B7] Guo S. Tao S. Yin W. Qi X. Sheng J. Zhang C. (2016). Exosomes from human synovial-derived mesenchymal stem cells prevent glucocorticoid-induced osteonecrosis of the femoral head in the rat. *Int. J Biol. Sci.* 12 1262–1271. 10.7150/ijbs.16150 27766040 PMC5069447

[B8] Jethwa S. A. Leah E. J. Zhang Q. Bright N. A. Oxley D. Bootman M. D. (2016). Exosomes bind autotaxin and act as a physiological delivery mechanism to stimulate LPA receptor signalling in cells. *J Cell Sci.* 129 3948–3957. 10.1242/jcs.184424 27557622 PMC5087657

[B9] Jin Z. Ren J. Qi S. (2020). Human bone mesenchymal stem cells-derived exosomes overexpressing microRNA-26a-5p alleviate osteoarthritis via down-regulation of PTGS2. *Int. Immunopharmacol.* 78:105946. 10.1016/j.intimp.2019.105946 31784400

[B10] Kim H. Yang G. Park J. Choi J. Kang E. Lee B. K. (2019). Therapeutic effect of mesenchymal stem cells derived from human umbilical cord in rabbit temporomandibular joint model of osteoarthritis. *Sci. Rep.* 9:13854. 10.1038/s41598-019-50435-2 PMC6761110 31554894

[B11] Knowlden S. Georas S. N. (2014). The autotaxin-LPA axis emerges as a novel regulator of lymphocyte homing and inflammation. *J Immunol.* 192 851–857. 10.4049/jimmunol.1302831 24443508 PMC3905607

[B12] Lee Y. H. Park H. K. Auh Q. S. Nah H. Lee J. S. Moon H. J. (2020). Emerging potential of exosomes in regenerative medicine for temporomandibular joint Osteoarthritis. *Int. J Mol. Sci.* 21:1541. 10.3390/ijms21041541 32102392 PMC7073204

[B13] Li C. Wang S. Xing Z. Lin A. Liang K. Song J. (2017). A ROR1-HER3-lncRNA signaling axis modulates the Hippo-YAP pathway to regulate bone metastasis. *Nat. Cell Biol.* 19 106–119. 10.1038/ncb3464 28114269 PMC5336186

[B14] Lu L. Zhang X. Zhang M. Zhang H. Liao L. Yang T. (2015). RANTES and SDF-1 are keys in cell-based therapy of TMJ osteoarthritis. *J Dent. Res.* 94 1601–1609. 10.1177/0022034515604621 26377571

[B15] Phinney D. G. Pittenger M. F. (2017). Concise review: MSC-derived exosomes for cell-free therapy. *Stem Cells* 35 851–858. 10.1002/stem.2575 28294454

[B16] Qin Y. Sun R. Wu C. Wang L. Zhang C. (2016). Exosome: a novel approach to stimulate bone regeneration through regulation of osteogenesis and angiogenesis. *Int. J Mol. Sci.* 17:712. 10.3390/ijms17050712 27213355 PMC4881534

[B17] Ryu J. M. Han H. J. (2015). Autotaxin-LPA axis regulates hMSC migration by adherent junction disruption and cytoskeletal rearrangement via LPAR1/3–dependent PKC/GSK3β/β–catenin and PKC/Rho GTPase pathways. *Stem Cells* 33 819–832. 10.1002/stem.1882 25376707

[B18] Tao S. Yuan T. Zhang Y. Yin W. Guo S. Zhang C. (2017). Exosomes derived from miR-140-5p-overexpressing human synovial mesenchymal stem cells enhance cartilage tissue regeneration and prevent osteoarthritis of the knee in a rat model. *Theranostics* 7 180–195. 10.7150/thno.17133 28042326 PMC5196895

[B19] Teo A. Q. A. Wong K. L. Shen L. Lim J. Y. Toh W. S. Lee E. H. (2019). Equivalent 10-year outcomes after implantation of autologous bone marrow-derived mesenchymal stem cells versus autologous chondrocyte implantation for chondral defects of the knee. *Am. J Sports Med.* 47 2881–2887. 10.1177/0363546519867933 31433674

[B20] Théry C. Witwer K. W. Aikawa E. Alcaraz M. J. Anderson J. D. Andriantsitohaina R. (2018). Minimal information for studies of extracellular vesicles 2018 (MISEV2018), a position statement of the international society for extracellular vesicles and update of the MISEV2014 guidelines. *J. Extracell Vesicles* 7:1535750. 10.1080/20013078.2018.1535750 PMC6322352 30637094

[B21] Vonk L. A. van Dooremalen S. F. J. Liv N. Klumperman J. Coffer P. J. Saris D. B. F. (2018). Mesenchymal stromal/stem cell-derived extracellular vesicles promote human cartilage regeneration in vitro. *Theranostics* 8 906–920. 10.7150/thno.20746 29463990 PMC5817101

[B22] Wang Y. Qin X. Zhu X. Chen W. Zhang J. Chen W. (2018). Oral Cancer-derived exosomal NAP1 enhances cytotoxicity of natural killer cells via the IRF-3 pathway. *Oral Oncol.* 76 34–41. 10.1016/j.oraloncology.2017.11.024 29290284 PMC11849059

[B23] Wang Y. Yu D. Liu Z. Zhou F. Dai J. Wu B. (2017). Exosomes from embryonic mesenchymal stem cells alleviate osteoarthritis through balancing synthesis and degradation of cartilage extracellular matrix. *Stem Cell Res.* 8:189. 10.1186/s13287-017-0632-0 PMC5556343 28807034

[B24] Witwer K. W. Van Balkom B. W. M. Bruno S. Choo A. Dominici M. Gimona M. (2019). Defining mesenchymal stromal cell (MSC)-derived small extracellular vesicles for therapeutic applications. *J. Extracell Vesicles* 8:1609206. 10.1080/20013078.2019.1609206 31069028 PMC6493293

[B25] Wu M. Cai J. Yu Y. Hu S. Wang Y. Wu M. (2021). Therapeutic agents for the treatment of temporomandibular joint disorders: progress and perspective. *Front. Pharmacol.* 11:596099. 10.3389/fphar.2020.596099 33584275 PMC7878564

[B26] Yu B. Zhang X. Li X. (2014). Exosomes derived from mesenchymal stem cells. *Int. J Mol. Sci.* 15 4142–4157. 24608926 10.3390/ijms15034142 PMC3975389

[B27] Zhang S. Chuah S. J. Lai R. C. Hui J. H. P. Lim S. K. Toh W. S. (2018). MSC exosomes mediate cartilage repair by enhancing proliferation, attenuating apoptosis and modulating immune reactivity. *Biomaterials* 156 16–27. 10.1016/j.biomaterials.2017.11.028 29182933

[B28] Zhang S. Teo K. Y. W. Chuah S. J. Lai R. C. Lim S. K. Toh W. S. (2019). MSC exosomes alleviate temporomandibular joint osteoarthritis by attenuating inflammation and restoring matrix homeostasis. *Biomaterials* 200 35–47. 10.1016/j.biomaterials.2019.02.006 30771585

