# Supplementary figures and images for: BMSC-Derived Small Extracellular Vesicles Induce Cartilage Reconstruction of Temporomandibular Joint Osteoarthritis via Autotaxin–YAP Signaling Axis
Source: Front Cell Dev Biol. 2021 Apr 1;9:656153. doi: 10.3389/fcell.2021.656153 (PMC8047210; doi:10.3389/fcell.2021.656153)

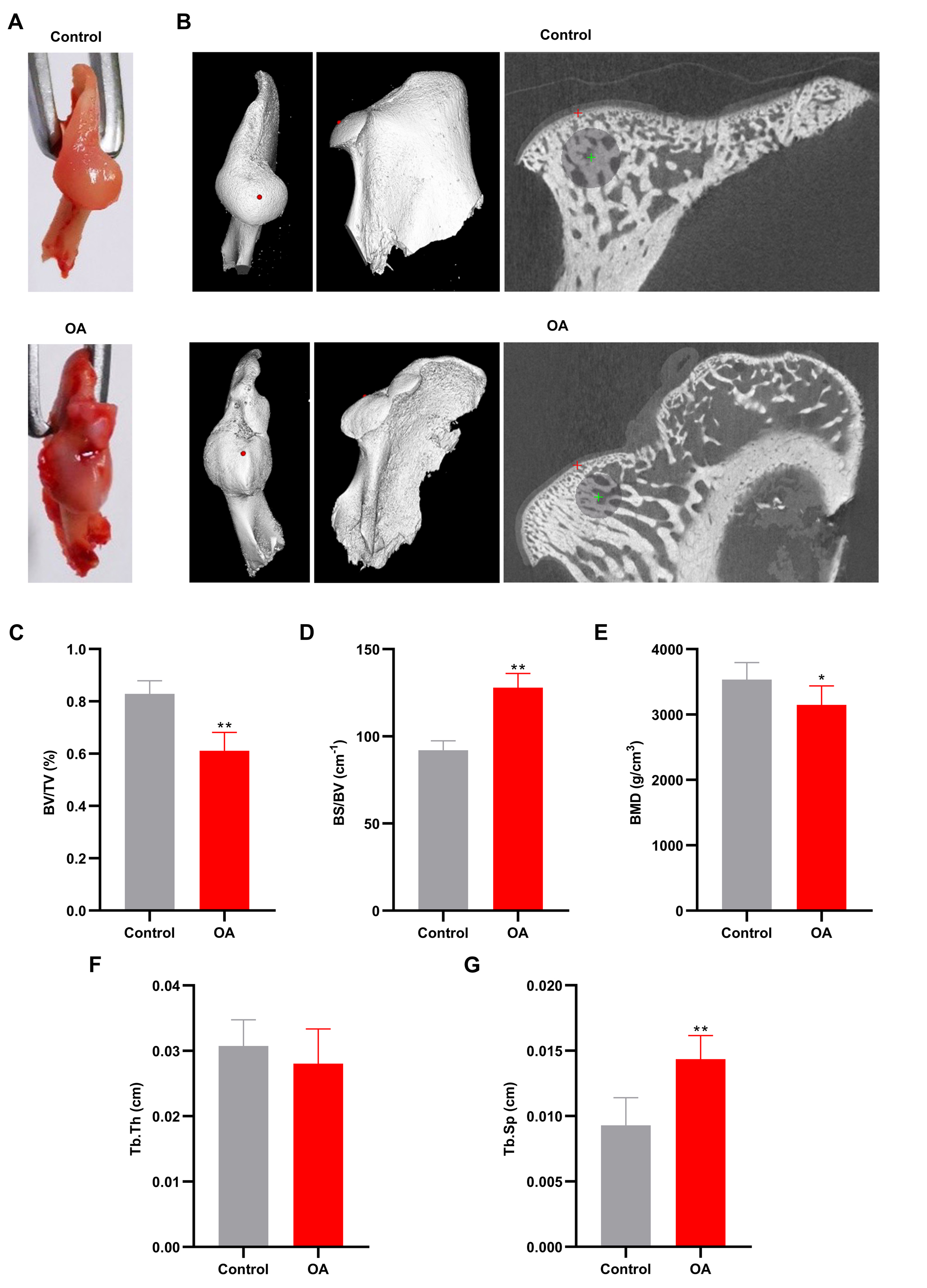

Supplement: Supplementary Figure 1 — Gross observation and Micro-CT of condyles of TMJOA animal model. (A) Gross observation of condyles of TMJOA animal model. (B) Micro-CT images of condyles of TMJOA animal model. (C–G). Parameters of micro-CT analysis of TMJOA animal model, including BVF, BS/BV, BMD, Tb.Th and Tb.Sp. ∗P < 0.05, ∗∗P < 0.01. [file Image_1.JPEG]

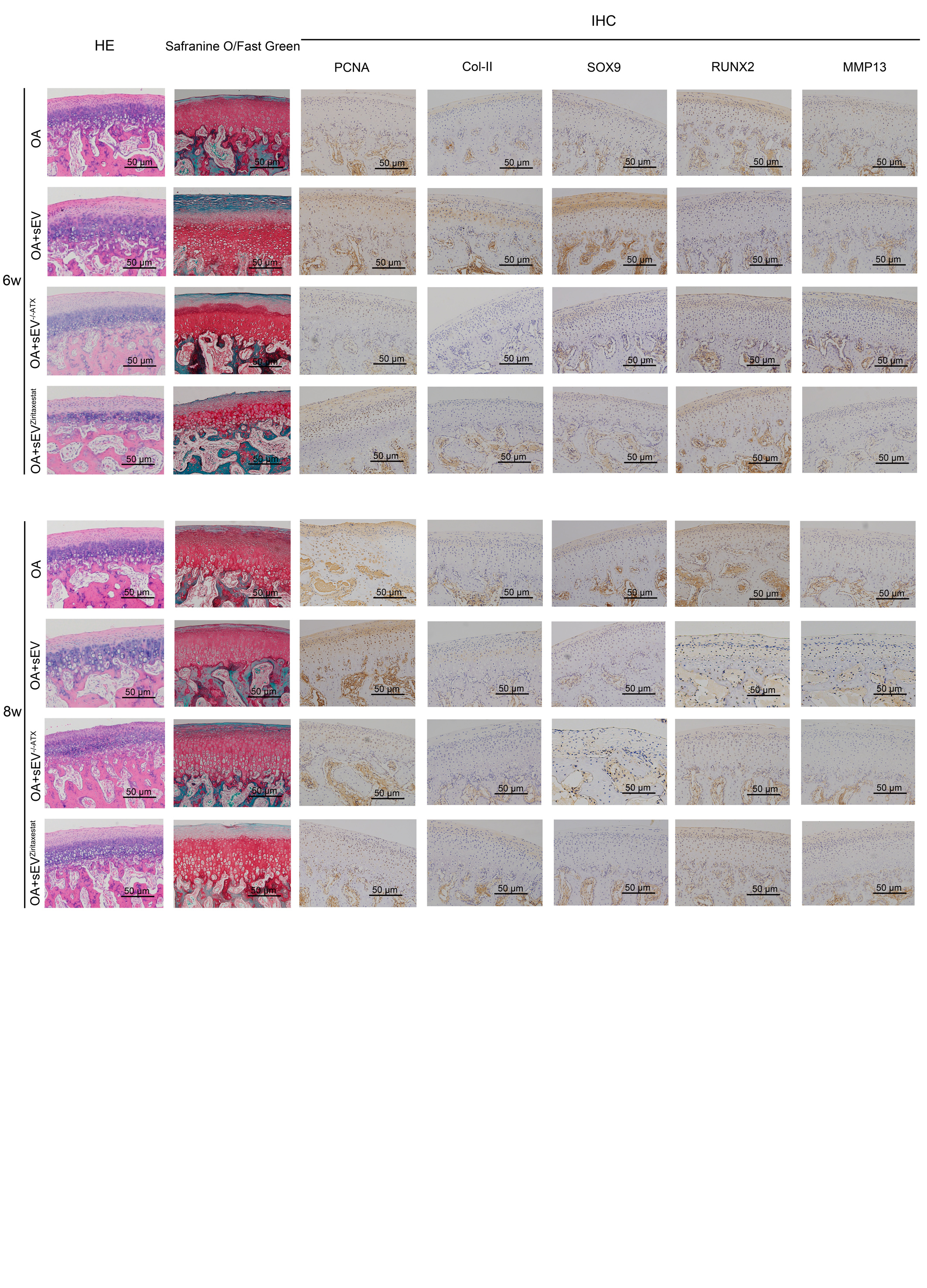

Supplement: Supplementary Figure 2 — Histologic and IHC analysis of condyles of TMJOA model treated with multiple kinds of BMSC-sEVs. HE, safranin O/fast green and IHC analysis of condyles of TMJOA animal model treated with BMSC-sEVs, normal and autotaxin knockdown or inhibition for 6 w and 8 w, respectively (200×). [file Image_2.JPEG]
