# Supplementary material for: BMSC-Derived Small Extracellular Vesicles Induce Cartilage Reconstruction of Temporomandibular Joint Osteoarthritis via Autotaxin–YAP Signaling Axis
Source: Front Cell Dev Biol. 2021 Apr 1;9:656153. doi: 10.3389/fcell.2021.656153 (PMC8047210; doi:10.3389/fcell.2021.656153)
Supplement: Supplementary file 3 [file Table_1.DOCX]

**Supplementary Table 1.** ICRS Visual Histological Assessment Scale

| **Feature** | **Score** |
| --- | --- |
| 1. **Surface** |  |
| Discontinuous | 0 |
| Continuous | 3 |
| 1. **Matrix** |  |
| Fibrous tissue | 0 |
| Fibrocartilage | 1 |
| Mixture | 2 |
| Hyaline | 3 |
| 1. **Cell distribution** |  |
| Disorganized | 0 |
| Clusters | 1 |
| Mixed | 2 |
| Columnar | 3 |
| 1. **Cell population viability** |  |
| Less than 10% viable | 0 |
| Partially viable | 1 |
| Predominantly viable | 3 |
| 1. **Subchondral bone** |  |
| Detached at base | 0 |
| Bone necrosis | 1 |
| Increased remodeling | 2 |
| Normal | 3 |
| 1. **Calcified cartilage** |  |
| Abnormal | 0 |
| Normal | 3 |

**Supplementary Table 2.** Wakitani Histological Grading Scale for the Defects of Cartilage

| **Feature** | **Score** |
| --- | --- |
| 1. **Cartilage cell morphology** |  |
| None only | 4 |
| Mostly none | 3 |
| Mostly fibrous | 2 |
| Mostly Hyaline | 1 |
| Hyaline | 0 |
| 1. **Metachromasia** |  |
| No metachromatic stain | 3 |
| Markedly reduced | 2 |
| Slightly reduced | 1 |
| Normal | 0 |
| 1. **Surface regularity** |  |
| Severely irregular | 3 |
| Irregular | 2 |
| Moderate | 1 |
| Smooth | 0 |
| 1. **Thickness of cartilage** |  |
| <1/3 | 2 |
| 1/3-2/3 | 1 |
| >2/3 | 0 |
| 1. **Integration of donor with host adjacent cartilage** |  |
| Neither edge integrated | 2 |
| One edge integrated | 1 |
| Both edges integrated | 0 |
| **Total Maximum** | **14** |
